# A genome-wide study of the lipoxygenase gene families in *Medicago truncatula* and *Medicago sativa* reveals that *MtLOX24* participates in the methyl jasmonate response

**DOI:** 10.1186/s12864-024-10071-1

**Published:** 2024-02-19

**Authors:** Lei Xu, Xiaoxi Zhu, Fengyan Yi, Yajiao Liu, Bilig Sod, Mingna Li, Lin Chen, Junmei Kang, Qingchuan Yang, Ruicai Long

**Affiliations:** 1 Institute of Animal Sciences, Chinese Academy of Agricultural Sciences, Beijing, 100193 China; 2 State Key Laboratory of Grassland Agro-Ecosystems, Key Laboratory of Grassland Livestock Industry Innovation, Ministry of Agriculture and Rural Affairs, Engineering Research Center of Grassland Industry, Ministry of Education, College of Pastoral Agriculture Science and Technology, Lanzhou University, Lanzhou, 730020 China; 3 Inner Mongolia Academy of Agricultural and Animal Husbandry Sciences, Hohhot, 010031 China

**Keywords:** *Medicago*, Lipoxygenase, Methyl jasmonate, *Arabidopsis thaliana*, Overexpression

## Abstract

**Background:**

Lipoxygenase (LOX) is a multifunctional enzyme that is primarily related to plant organ growth and development, biotic and abiotic stress responses, and production of flavor-associated metabolites. In higher plants, the LOX family encompasses several isozymes with varying expression patterns between tissues and developmental stages. These affect processes including seed germination, seed storage, seedling growth, fruit ripening, and leaf senescence. *LOX* family genes have multiple functions in response to hormones such as methyl jasmonate (MeJA) and salicylic acid.

**Results:**

In this study, we identified 30 and 95 *LOX* homologs in *Medicago truncatula* and *Medicago sativa*, respectively. These genes were characterized with analyses of their basic physical and chemical properties, structures, chromosomal distributions, and phylogenetic relationships to understand structural variations and their physical locations. Phylogenetic analysis was conducted for members of the three *LOX* subfamilies (9-LOX, type I 13-LOX, and type II 13-LOX) in *Arabidopsis thaliana*, *Glycine max*, *M. truncatula*, and *M. sativa*. Analysis of predicted promoter elements revealed several relevant *cis*-acting elements in *MtLOX* and *MsLOX* genes, including abscisic acid (ABA) response elements (ABREs), MeJA response elements (CGTCA-motifs), and antioxidant response elements (AREs). *Cis*-element data combined with transcriptomic data demonstrated that *LOX* gene family members in these species were most likely related to abiotic stress responses, hormone responses, and plant development. Gene expression patterns were confirmed via quantitative reverse transcription PCR. Several *MtLOX* genes (namely *MtLOX15*, *MtLOX16*, *MtLOX20*, and *MtLOX24*) belonging to the type I 13-LOX subfamily and other *LOX* genes (*MtLOX7*, *MtLOX11*, *MsLOX23*, *MsLOX87*, *MsLOX90*, and *MsLOX94*) showed significantly different expression levels in the flower tissue, suggesting roles in reproductive growth. Type I 13-LOXs (*MtLOX16*, *MtLOX20*, *MtLOX21*, *MtLOX24, MsLOX57*, *MsLOX84*, *MsLOX85*, and *MsLOX94*) and type II 13-LOXs (*MtLOX5*, *MtLOX6*, *MtLOX9*, *MtLOX10*, *MsLOX18*, *MsLOX23*, and *MsLOX30*) were MeJA-inducible and were predicted to function in the jasmonic acid signaling pathway. Furthermore, exogenous *MtLOX24* expression in *Arabidopsis* verified that *MtLOX24* was involved in MeJA responses, which may be related to insect-induced abiotic stress.

**Conclusions:**

We identified six and four *LOX* genes specifically expressed in the flowers of *M. truncatula* and *M. sativa*, respectively. Eight and seven *LOX* genes were induced by MeJA in *M. truncatula* and *M. sativa*, and the *LOX* genes identified were mainly distributed in the type I and type II 13-LOX subfamilies. *MtLOX24* was up-regulated at 8 h after MeJA induction, and exogenous expression in *Arabidopsis* demonstrated that *MtLOX24* promoted resistance to MeJA-induced stress. This study provides valuable new information regarding the evolutionary history and functions of *LOX* genes in the genus *Medicago*.

**Supplementary Information:**

The online version contains supplementary material available at 10.1186/s12864-024-10071-1.

## Background

Lipoxygenase (LOX) is a dioxygenase-containing non-heme iron that catalyses unsaturated fatty acids with linoleic acid (LA), α-linolenic acid and arachidonic acid as substrates. It is present in both plants and animals, but its catalytic processes differ between kingdoms. In animals, LOX catalyzes the formation of neuroprotectin from arachidonic acid (20 carbon) and eicosapentaenoic acid [1]. Substrates in other animals include linolenic acid and docosahexaenoic acid (DHA). In plants, LOX catalyzes the dehydrogenation and oxygenation of unsaturated fatty acids such as linoleic acid, linolenic acid, and arachidonic acid to form hydroperoxides. Subsequent specialized metabolic reactions form oxygen-containing derivatives such as the plant hormone jasmonic acid (JA), reactive oxylipids with epoxides, conjugated carbonyls or aldehydes, and antibacterial and antifungal compounds, such as leaf aldehydes or diethylene ethers [2–5]. In addition, some polyunsaturated fatty acids can be catalyzed by α-dioxygenase to form α-hydroxy polyunsaturated fatty acids (PUFAs) or α-peroxy PUFAs [6, 7].

Since the 1930s, studies have shown that soybean flavor formation is related to enzymatic reactions of unsaturated fatty acids. The key enzyme in these oxidation reactions is LOX [8]. Later studies showed that LOX is widespread among fungi, bacteria, algae, and protozoa, and is more prevalent in plants than in animals [9]. To date, several *LOX* genes have been cloned in model plants such as *Arabidopsis thaliana* [10], rice, wheat, barley, maize, and tomato [11]. Furthermore, these genes show high activity levels in legumes.

Members of the LOX family can be classified based on their optimal pH, subcellular localization, and reaction location for specific substrates. Most researchers divide the LOX family into 9-LOXs and 13-LOXs based on the reaction sites of the catalytic substrates linoleic acid (LA, 18:2) and linolenic acid (LeA, 18:3) during oxidation; enzymatic PUFA oxidation can occur on carbon 9 (9-LOX) or carbon 13 (13-LOX) [12]. Furthermore, 13-LOX proteins can be further divided into two subgroups, type I and type II. Type I 13-LOX proteins have no transport peptides, show high sequence similarity (> 75%) with each other, and are usually localized to the cytoplasm. Type II 13-LOX proteins are localized to the chloroplast by an N-terminal transport peptide and show only moderate sequence similarity (> 35%) between subfamily members [13]. In animals, LOXs can be divided into the 5-LOX, 8-LOX, 9-LOX, 11-LOX, 12-LOX, and 15–1-LOX subfamilies based on the insertion position of molecular oxygen for oxygenation reactions [14]. Numerous studies have reported the presence of *LOX* family genes in plant species. To date, six *LOX*s have been reported in *Arabidopsis* [10], 23 in Chinese white pear (*Pyrus bretschneideri*) [15], 18 in melon (*Cucumis melo*) [16], 36 in soybean (*Glycine max*), 28 in *Medicago truncatula*, 10 in *Cicer arietinum*, five in *Lotus japonicus* [17], 14 in rice [18], 64 among four cotton species (*Gossypium hirsutum*, *Gossypium barbadense*, *Gossypium arboretum*, and *Gossypium raimondii*) [19], 41 among three banana species (*Musa acuminata*, *Musa balbisiana*, and *Musa itinerans*) [20], 14 in diploid woodland strawberry (*Fragaria vesca*) [21], and 20 in *Artemisia annua* [22]. Some researchers have shown that genes in the 9-LOX subfamily play important roles in seed, organ, and fruit development. They also function in defense responses against pathogens such as *Phytophthora parasitica varocotianae* (PPN) in tobacco [23], yellow branch mold in tomato [24] and *Phytophthora infestans* in potato [25]. Genes in the 13-LOX subfamily have key roles in plant development, senescence, and biotic stress responses (such as pathogen infection and insect herbivory) [17, 26, 27]. These genes also have essential roles in responses to hormones, including salicylic acid (SA), methyl jasmonate (MeJA), and abscisic acid (ABA) [21]. Most soybean *LOX* genes in the 13-LOX subfamilies are induced by parasitic nematodes and exhibit crosstalk with other signaling networks, such as the ethylene and SA pathways. They may also interact with members of other gene families, such as WRKYs [28]. In summary, genes in different LOX subfamilies within a species may have similar or opposing functions and show differential expression between tissues. Similarly, *LOX* genes in the same subfamily may also have distinct functions and spatiotemporal expression patterns.

LOX is known to contribute to biosynthesis of oxylipins and JA, both of which mediate the JA signaling pathway and play important roles in plant development and defense against biotic stressors [29]. Exogenous JA induces plant defense responses in a concentration-dependent manner. Low concentrations of MeJA can promote plant growth, induce JA synthesis, enhance expression of downstream defense-related genes, and alleviate stress-induced damage. However, high concentrations of MeJA and oxylipin inhibit seed or pollen germination [30, 31], which may be due to inhibitory effects of jasmonate on root meristem recombination, cell division, cell elongation, and cell precocity [31]. MeJA treatment can prolong *A. thaliana* flowering under short-day conditions [32]. MeJA-induced damage can also simulate insect defense responses [33]. This may be due to the influence of exogenous JA content, levels of volatile terpenes (e.g., α-terpene, α-pinene, β-phellandrene, β-caryophyllene, and myrcene) and specialized metabolites, such as flavonoids and phenolic compounds [34]. In *Dunaliella salina*, wheat, and tomato, exogenous MeJA treatment causes increases in β-carotene content and chlorophyll degradation, but reductions in the respiratory and photosynthetic rates [35]. MeJA treatment can increase peroxidase (POD) and superoxide dismutase (SOD) activity and enhance the plant capacity for scavenging active oxygen. Increased treatment time is associated with decreased SOD and POD activities, leading to active oxygen damage and enhanced membrane lipid peroxidation [36]. In simulated pest experiments with JA, SA has been shown to antagonize the JA signaling pathway [37, 38].

We here sought to characterize the *LOX* gene families in *M. truncatula* and *M. sativa*. Genome-wide analysis revealed 30 *MtLOX* and 95 *MsLOX* genes, which were comprehensively characterized via analyses of the gene structures, chromosomal distributions, phylogenetic relationships, promoter *cis*-elements, and expression patterns. We also determined the function of *MtLOX24* during MeJA treatment based on exogenous expression in *Arabidopsis*. Our findings provide valuable insights regarding the complex network and functionality of *LOX* genes in legumes.

## Results

### Identification of LOX family members in *M. truncatula* and *M. sativa* and basic physicochemical properties analysis

We here identified 30 and 95 *LOX* genes in *M. truncatula* and *M. sativa* (cv. ‘Zhongmu No. 4’), respectively, and referred to them as *MtLOX 1*–*30* and *MsLOX 1*–*95*, respectively (Additional file 1: Table S1; Additional file 2: Table S2). The lengths of the predicted products encoded by these genes ranged from 174 amino acids (aa) (*MtLOX8*) to 927 aa (*MtLOX27*) in *M. truncatula*, and from 248 aa (*MsLOX65*) to 2892 aa (*MsLOX40*) in *M. sativa*. The average length among all *Medicago* LOXs was 830 aa. The predicted MtLOX and MsLOX proteins varied in molecular weight from 20.56–104.81 kDa and from 28.29–324.36 kDa, respectively. The theoretical isoelectric points ranged from 5.3 (MtLOX15) to 8.87 (MtLOX14) in *M. truncatula* and from 5.12 (MsLOX77) to 7.99 (MsLOX1) in *M. sativa*. The average Grand Average of HYdropathicity (GRAVY) values of both the MtLOXs and the MsLOXs were negative, indicating that the proteins were primarily hydrophilic. Among the MtLOXs, eight were predicted to be localized to only the chloroplast, 17 to only the cytoplasm, and five to both the chloroplast and the cytoplasm. Among the MsLOX proteins, 30 were predicted to be localized to only the chloroplast, two to both the mitochondrion and the cytoplasm, 14 to both the chloroplast and the cytoplasm, and the remaining to the cytoplasm (Additional file 1: Table S1, Additional file 2: Table S2).

### Chromosomal distribution of *MtLOX *and *MsLOX* genes

*MtLOX* genes were distributed on seven of the eight chromosomes in both *M. truncatula* and *M. sativa* (Additional file 3: Figure S1 A, and B). Chromosome (chr) 08 contained the largest number of *MtLOX* genes (17), followed by chr03 (five). There were only two *MtLOX* genes each on chr01, chr02, and chr04. Chr05 and chr07 contained only one *MtLOX* each, and there was no *MtLOX* gene on chr06. Chr08 also contained the largest number of *LOX* genes in *M. sativa* (42 genes, 44.21%), followed by chr03 (12 genes, 12.63%), chr04 (11 genes, 16.92%), chr01 (nine genes, 13.85%), and chr05 (seven genes, 10.77%). Many fewer genes were distributed on chr07 (four genes, 4.21%), chr02 (three genes, 3.16%), and chr06 (two genes, 2.11%). Several *MsLOX* genes were distributed on the four *M. sativa* homologous chromosomes, and four *MsLOX*s (namely *MsLOX92*, *MsLOX93*, *MsLOX94*, and *MsLOX95*) were identified in scaffolds.

### Phylogenetic analysis of *MtLOX* and *MsLOX* genes

**Fig. 1:**
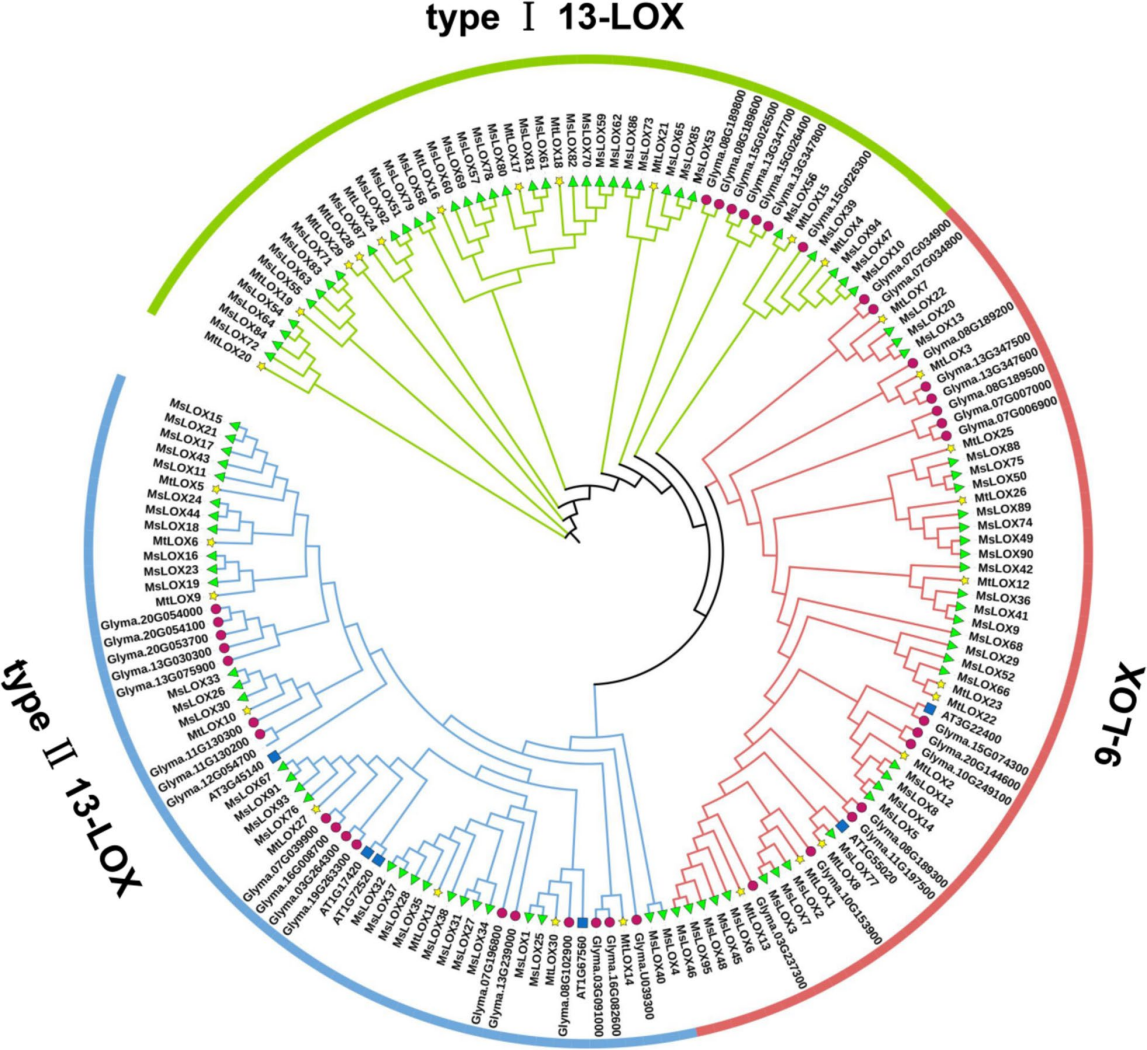
Phylogenetic analysis of LOX proteins in *Arabidopsis thaliana*, *Glycine max*, *Medicago truncatula*, and *Medicago sativa.* The phylogenetic tree was generated from six AtLOXs, 40 GmLOXs, 30 MtLOXs, and 111 MsLOXs. The branch colors represent LOX subgroups. The color and shape at the end of each branch represents the species: *Arabidopsis* (blue square), soybean (purple circle), *M. truncatula* (yellow five-pointed star), or *M. sativa* (green triangle)

To understand the evolutionary relationships between *LOX* genes, a phylogenetic tree was constructed from LOX proteins encoded by *M. truncatula*, *M. sativa*, *G. max*, and the model plant *Arabidopsis* (Fig. 1). *LOX* family genes were categorized as 9-LOXs or 13-LOXs based on their classification in *Arabidopsis*, which depended on the reaction site of the catalytic substrates linoleic acid (LA, 18:2) and linolenic acid (LeA, 18:3) during oxidation [12]. Among them, all 9-LOXs amino acid sequences account for about a third of all amino acid sequences (Fig. 1, red colour), and 9-LOX differentiation occurs between the differentiation of type I 13-LOXs (Fig. 1, green colour) and type II 13-LOXs (Fig. 1, blue colour). Furthermore, 13-LOXs were divided into type I and type II based on their phylogenetic relationships in *G. max*. There were 11, 11, and eight 9-LOXs, type I 13-LOXs, and type II 13-LOXs, respectively, in *M. truncatula* and 30, 34, and 31 members of the same subfamilies, respectively, in *M. sativa* (Table 1, Additional file 4: Table S3, Additional file 5: Table S4).

**Table 1:** Distribution of genes among *LOX* subfamilies in four species

Subfamily	*Arabidopsis*	*G. max*	*M. truncatula*	*M. sativa*
9-LOX	2	16	11	30
Type I 13-LOX	0	7	11	34
Type II 13-LOX	4	17	8	31
Total	6	40	30	95

### Structural and motif analyses of *MtLOX* and *MsLOX* genes

**Fig. 2:**
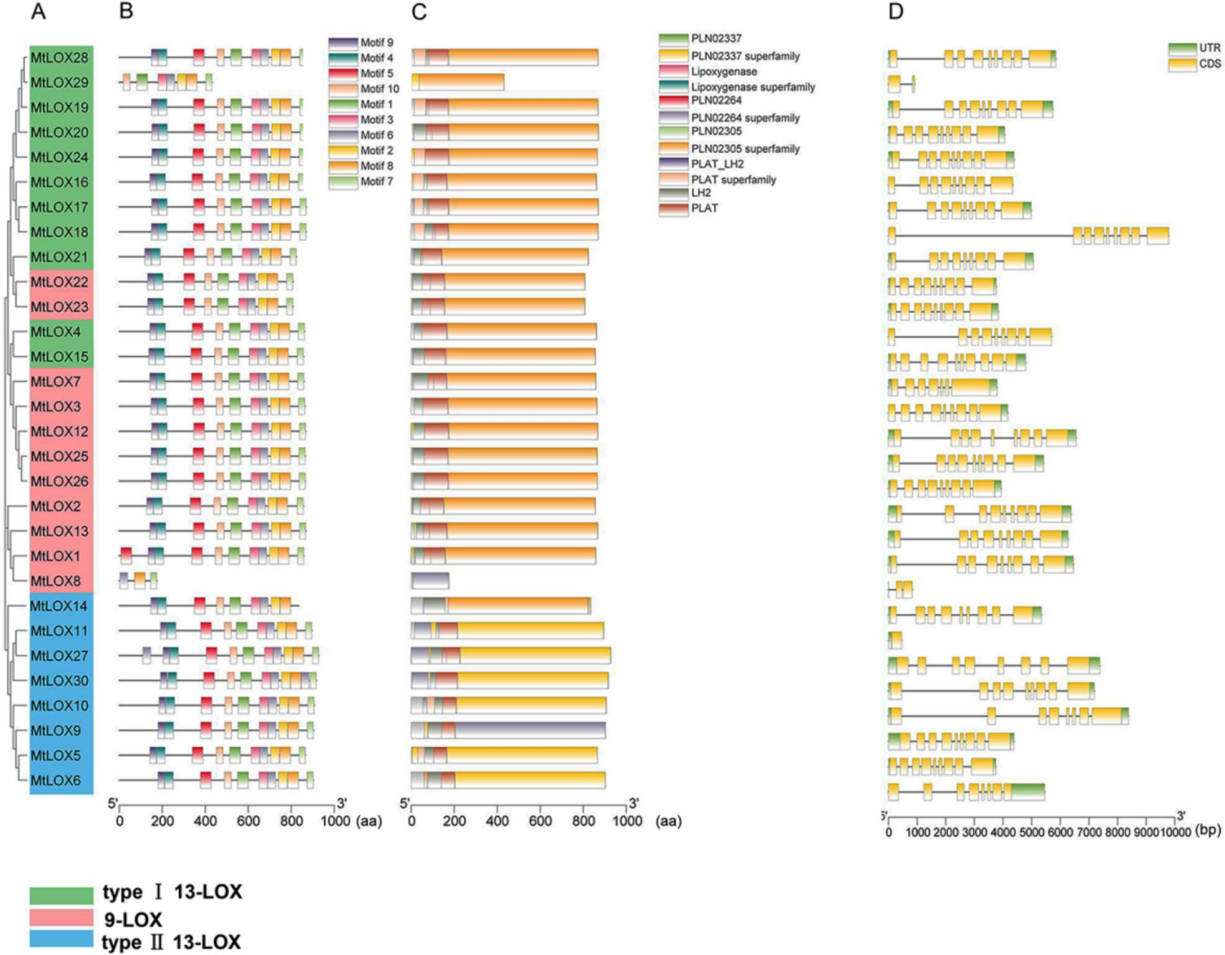
Gene structural analysis of *Medicago truncatula LOX*s. **A** Neighbor-joining phylogenetic tree showing relationships between *MtLOX* genes. **B** Motif analysis with the MEME suite yielded 10 motifs, each represented by a different color. **C** Conserved protein domain analysis with NCBI CDD predicted 12 conserved domains, each represented by a different color. **D** Exon–intron structural analysis. Yellow indicates exons, gray indicates introns, and green indicates untranslated regions (UTRs)

**Fig. 3:**
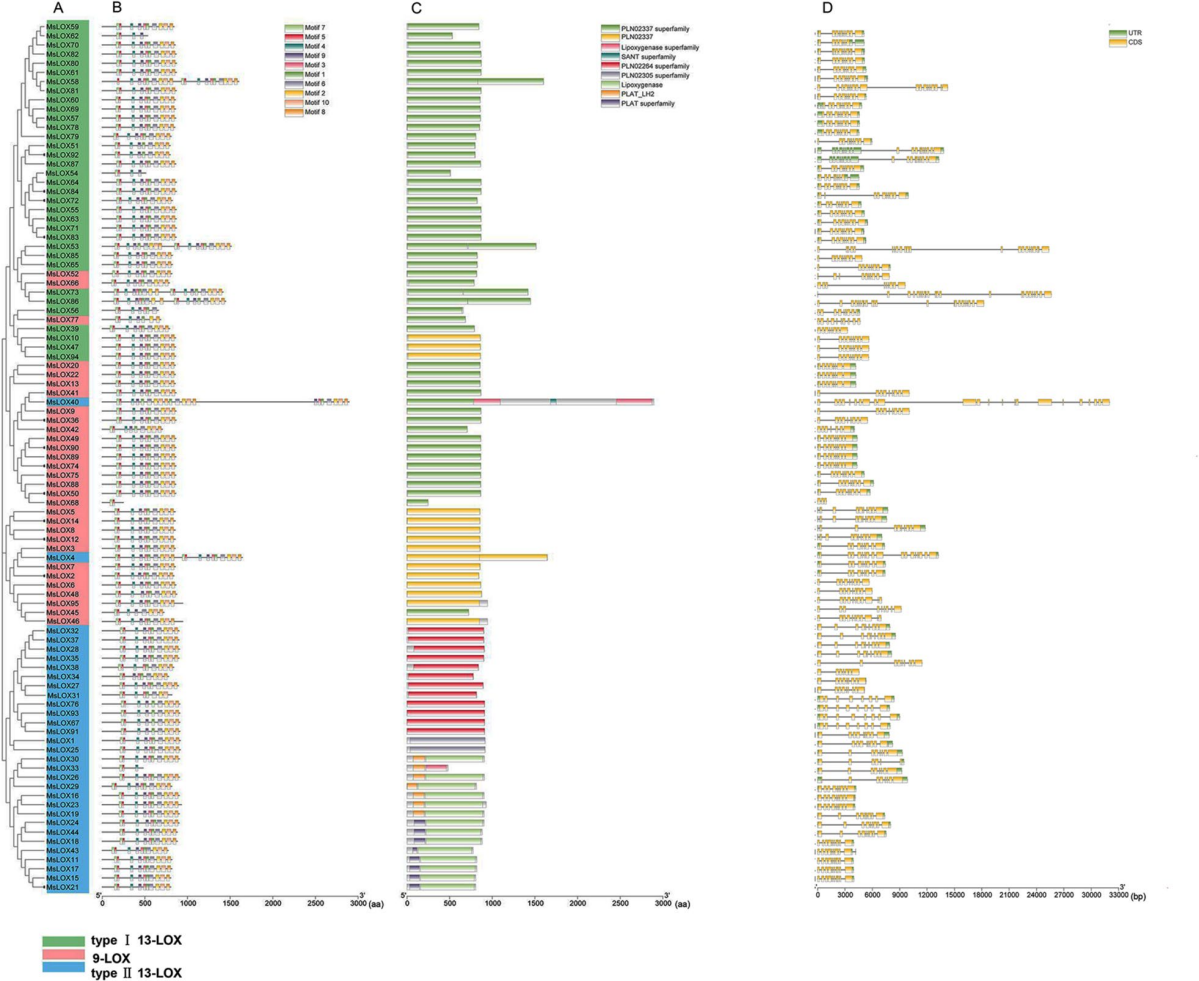
Gene structural analysis of *Medicago sativa LOX*s. **A** Neighbor-joining phylogenetic tree showing relationships between *MsLOX* genes. **B** Motif analysis with the MEME suite yielded 10 motifs, each represented by a different color. **C** Conserved protein domain analysis with NCBI CDD predicted 12 conserved domains, each represented by a different color. **D** Exon–intron structural analysis. Yellow indicates exons, gray indicates introns, and green indicates untranslated regions (UTRs)

Evolutionary relationships between *LOX* family genes were next analyzed in *M. truncatula* and *M. sativa* based on predicted protein motifs (Figs. 2A, 3A). A total of 10 conserved motifs were identified in the LOXs. The distribution of conserved motifs was consistent with the relationships between genes depicted in the phylogenetic tree (Figs. 2B, 3B). Most MtLOXs contained 10 motifs, except four proteins: MtLOX1, MtLOX8, MtLOX27, and MtLOX29. MtLOX29 had lost motifs 9, 4, and 5, whereas MtLOX1 and MtLOX27 had gained additional copies of motifs 5 and 6, respectively. Three motifs were identified in MtLOX8. In *M. sativa*, all 9-LOXs contained 10 or fewer gene motifs. For example, MsLOX77 lacked motifs 3, 6, and 10, and MsLOX42 lacked motif 3. MsLOX45 lacked motif 3 and motif 1, and MsLOX68 had only two motifs. The type I 13-LOX proteins MsLOX62 and MsLOX54 had four motifs each, and MsLOX56 had seven motifs. The type II 13-LOXs MsLOX53, MsLOX58, MsLOX73, MsLOX86, and MsLOX4 had another nine motifs, MsLOX40 had another seven motifs, and MsLOX33 had three motifs. The conserved residues in MtLOXs and MsLOXs are shown in Additional file 6: Figure S2.

Conserved structural domains were next investigated in the putative LOX proteins in *M. truncatula* and *M. sativa* (Figs. 2C, 3C; Additional file 7: Table S5; Additional file 8: Table S6). Most of the *LOX* family genes encoded PLAT (Pfam01477) and LOX (Pfam00305) domains. Members of the 9-LOX subfamily and the type I 13-LOX subfamily had similar conserved domains; most contained 13 domains, including PLN02337, LOX, PLN02264, PLN02305, PLAT_LH2, and PLAT. However, MtLOX29 and MtLOX8 lacked PLAT-related domains. Most type II 13-LOX subfamily proteins lacked one PLAT domain, except MtLOX14 and MsLOX38, which lacked two PLAT domains. MsLOX67, MsLOX76, MsLOX91, and MsLOX93 contained all 13 domains. MsLOX15, MsLOX17, MsLOX23, and MsLOX43 also had PRK06075 and complex1_49kDa superfamily (CL21493) domains. MsLOX11 had several additional domains: PRK06075, Complex1_49kDa superfamily, NuoD (COG0649), and NuoD (CL43223) SUPE domains.

Exon–intron structural analysis showed similar structures for most of the *MtLOX* genes, with eight or nine exons and seven or eight introns (Fig. 2D). However, *MtLOX8*, *MtLOX11*, and *MtLOX29* contained only one or two exons each. The majority of *MsLOX* genes had between eight and 23 exons and seven or eight introns, although *MsLOX68* contained only three exons (Fig. 3D). Most 9-LOX *MsLOX* genes contained eight or nine exons, similar to *MtLOX* genes*.* These results further indicated that structural evolution among members of the 13-LOX subfamily occurred later than that of 9-LOX subfamily members, and that the structures of 9-LOX genes were more highly conserved.

### *Cis*-acting regulatory element analyses of *MtLOX *and* MsLOX* genes

**Fig. 4:**
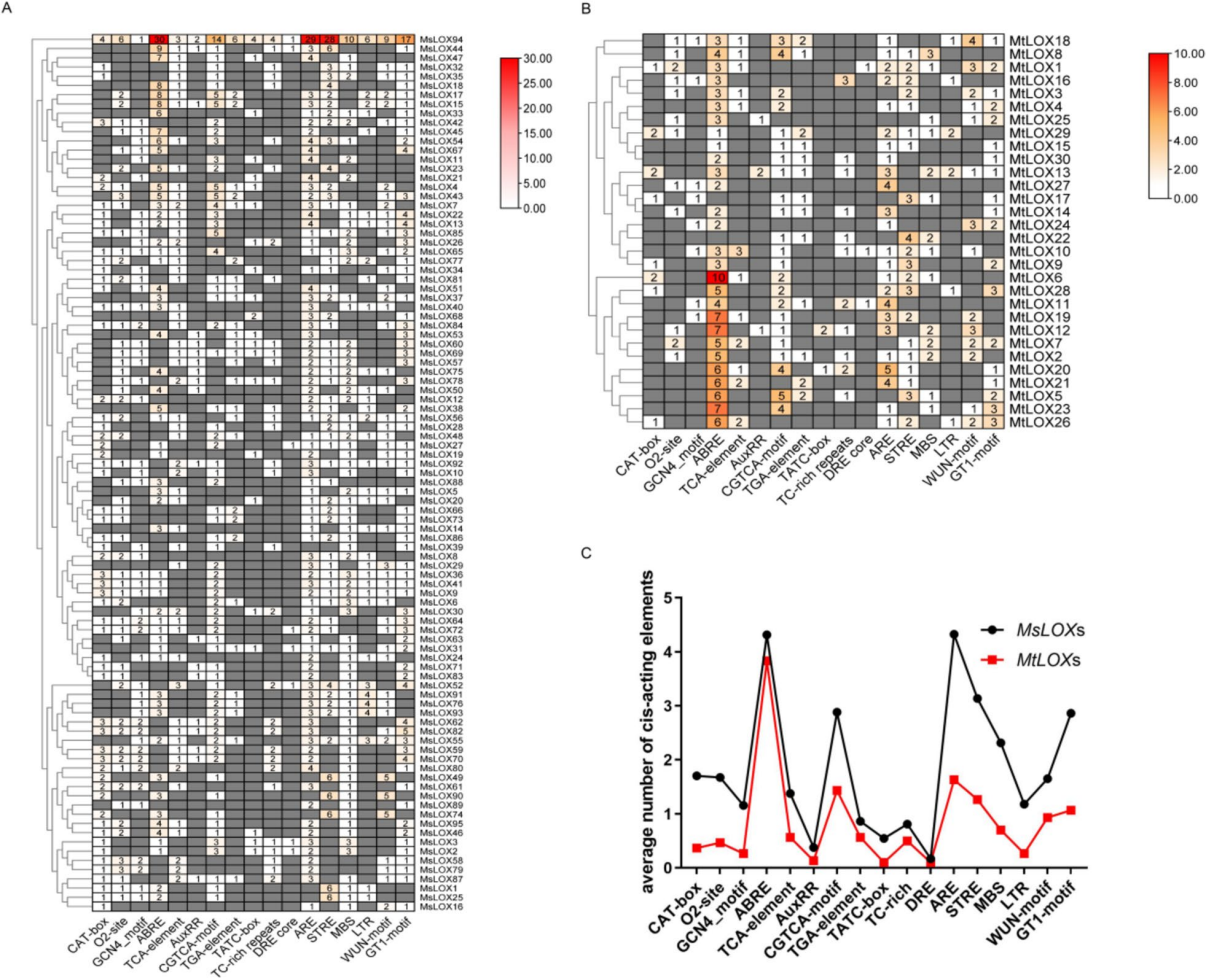
Promoter analysis of *LOX* genes in *Medicago truncatula* and *Medicago sativa.* **A** The number of *cis*-acting elements in the 2-kb promoter region upstream of the translation start site of each *MsLOX* gene. **B** The number of *cis*-acting elements in the 2-kb promoter region upstream of the translation start site of each *MtLOX* gene. **C** Distribution of specific types of *cis*-acting elements in *MtLOX* and *MsLOX* genes

To explore regulatory mechanisms associated with *LOX* gene expression, the 2-kb region upstream of the start codon for each *MtLOX* and *MsLOX* gene was analyzed to identify putative *cis*-acting regulatory elements. Most of these regulatory elements were related to plant development, phytohormone responses, and biotic and abiotic stress responses (Fig. 4). A large number of elements associated with hormone responses were present, specifically ABA response elements (ABREs) and MeJA response elements (CGTCA-motifs). In *M. sativa*, genes in the type II 13-LOX subfamily contained more ABREs than those in the other two subfamilies (Fig. 4A), but this was not the case in *M. truncatula* (Fig. 4B). *MtLOX* and *MsLOX* also contained stress-responsive *cis*-elements, including antioxidant response elements (AREs), stress response elements (STREs), and GT1 elements (GAAAAA). The promoters of these genes also contained several elements related to plant growth and development, including myeloblastosis (MBS) *cis*-elements, long terminal repeat (LTR) motifs, and wound-responsive elements (WUN-motifs); each gene contained three or fewer of these elements.

### MtLOX protein–protein interaction network analysis

A protein–protein interaction network was constructed for MtLOX proteins using the STRING database (Additional file 9: Figure S3 A). The network consisted of 24 edges (interactions) between 14 nodes (MtLOX proteins). Twelve MtLOXs were predicted to interact with MtLOX27 (AET01784) and with MtLOX14 (AET01336). STRING was further used to analyze a related network cluster (CL: 26,390), incorporating non-LOXs in the analysis; this produced a network consisting of 27 proteins (21 MtLOXs) with 133 interactions (Additional file 9: Figure S3 B). All MtLOXs were predicted to interact with AET05548, which was annotated as a pathogen-independent alpha dioxygenase. A 9- or 13-hydroperoxide lyase, hpl1 (AES89057), interacted with HPL3 (AES67192), MtLOX14 (AET01336), MtLOX27 (AET01784), and AES85033. HPL3, a 13-hydroperoxide lyase, interacted with MtLOX2 (AES62661), MtLOX5 (AES71573), MtLOX13 (AES82497), MtLOX14 (AET01336), and AES85033.

### *MtLOX* expression profile analysis

Expression profiles for 26 *MtLOX* genes were downloaded from the *M. truncatula* Gene Expression Atlas (Additional file 10: Figure S4; Additional file 11: Table S7). Six *MtLOX*s (*MtLOX10*, *MtLOX24*, *MtLOX25*, *MtLOX26*, *MtLOX27*, and *MtLOX30*) were expressed three to six times higher in the leaves, petioles, stems, vegetative buds (Vegbuds), flowers, and pods than in the roots or seeds. *MtLOX5*, *MtLOX9*, and *MtLOX16* were expressed more highly in the reproductive organs than in the vegetative organs, and *MtLOX5* was most highly expressed in the flowers. In contrast, *MtLOX19* was expressed at lower levels in the flowers and pods than in the petioles and stems. Seven *MtLOX* genes (*MtLOX19*, *MtLOX20*, *MtLOX28*, *MtLOX22*, *MtLOX21*, *MtLOX12*, and *MtLOX15*) had high expression levels at multiple nodule stages (Nod_4dpi and Nod_14dpi), RT_LCM_adjacent, RT_LCM_arbuscular, and RT_LCM_cortical. Only *MtLOX3* and *MtLOX4* were expressed specifically in the seeds, in which they were expressed five times higher than the other *MtLOX* genes.

### *MtLOX* and *MsLOX* spatial expression patterns and exogenous MeJA responses

**Fig. 5:**
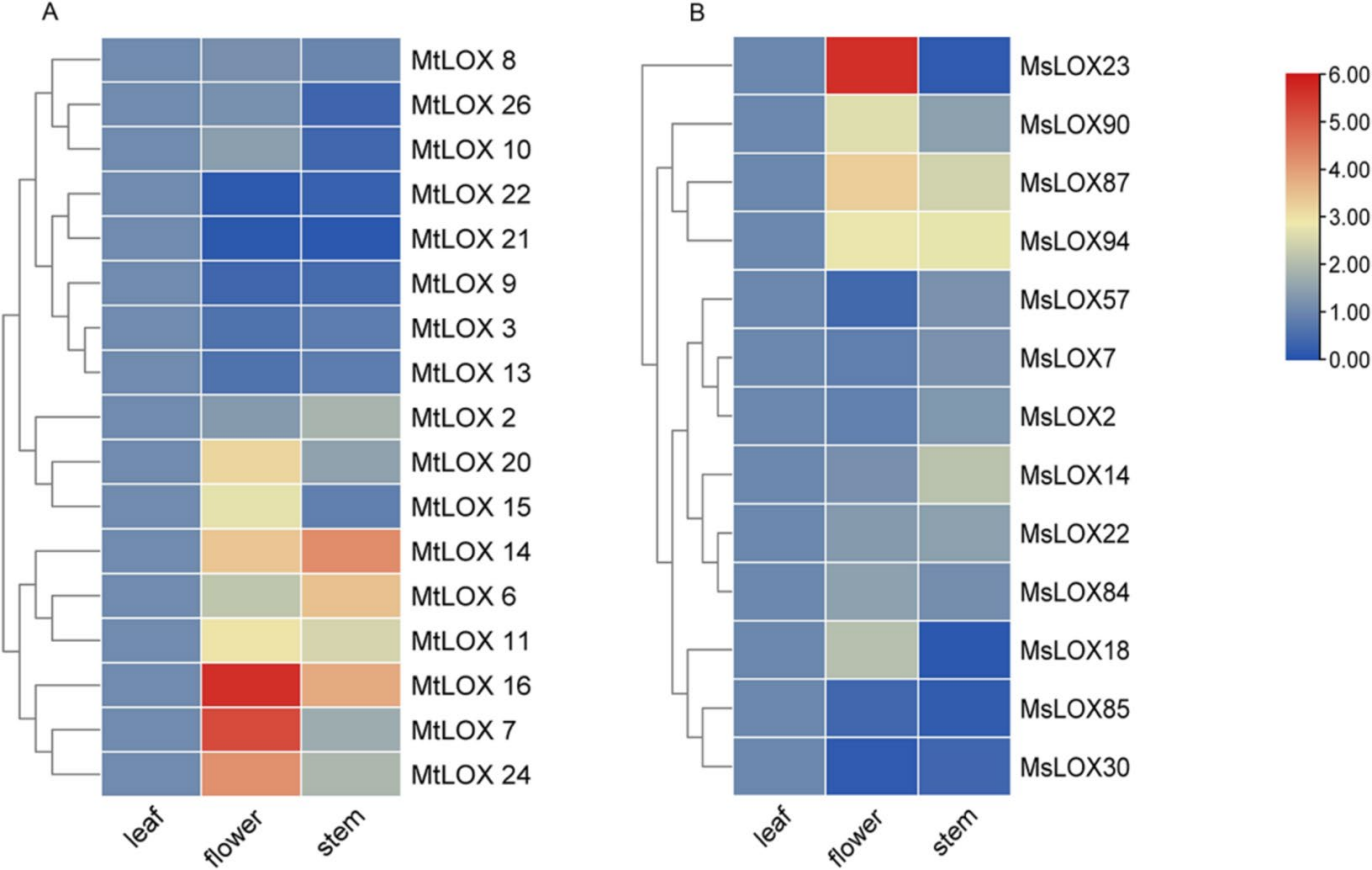
Expression patterns of *LOX* genes in different tissues of *Medicago truncatula* and *Medicago sativa.* Relative expression levels of *MtLOX* (**A**) and *MsLOX* (**B**) genes in the leaves, flowers, and stems as determined with qRT-PCR. Expression levels of *MtLOX* and *MsLOX* genes were normalized to levels of *MtActin* and *MsActin*, respectively. Hierarchical gene clustering was performed with the normalization method “standard score” on log_2_ transformed date. Colors indicates changes in expression compared to the leaf tissue, with red indicating an increase and blue indicating a decrease in expression

Quantitative reverse transcription (qRT)-PCR was used to confirm expression levels of *MtLOX* and *MsLOX* genes in the leaves, flowers, and stems of *M. truncatula* and *M. sativa* (Fig. 5). Several genes showed organ-specific high expression levels. For example, six *MtLOX* genes (*MtLOX*7, *MtLOX11*, *MtLOX15*, *MtLOX16*, *MtLOX20*, and *MtLOX24*) and four *MsLOX* genes (*MsLOX23*, *MsLOX87*, *MsLOX90*, and *MsLOX94*) were highly expressed in the flowers, suggesting roles in reproductive growth. Two *MtLOX* genes (*MtLOX14* and *MtLOX*16) and two *MsLOX* genes (*MsLOX87* and *MsLOX*94) were more highly expressed in the flowers and stems than in the leaves. Two *MtLOX* genes (*MtLOX*6 and *MtLOX*14) were highly expressed in the stem. *MtLOX9*, *MtLOX21, MtLOX22, MsLOX30*, and *MsLOX85* were more highly expressed in the leaves than in the other two tissues. Other *MtLOX* genes showed similar expression patterns.

**Fig. 6:**
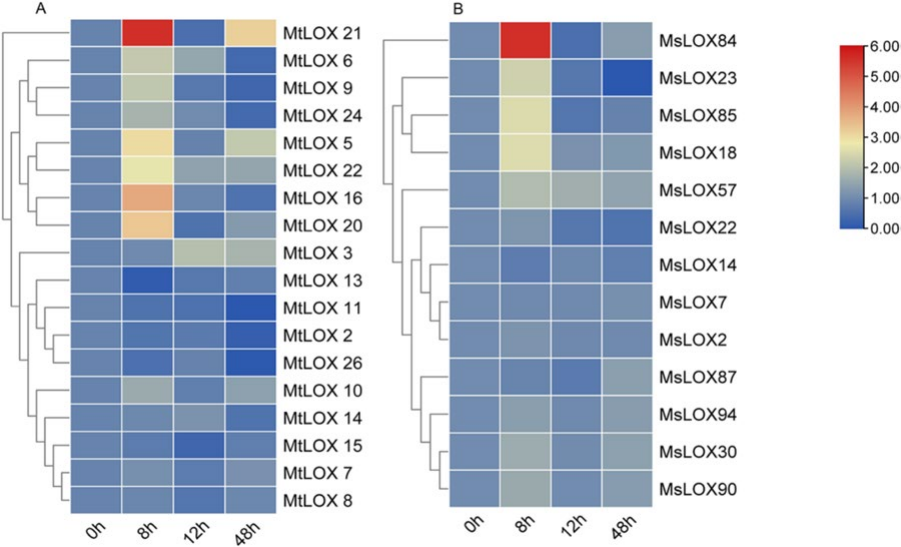
Expression patterns of *LOX* genes in *Medicago truncatula* and *Medicago sativa* after exogenous methyl jasomate (MeJA) treatment. Relative expression levels of *MtLOX* (**A**) and *MsLOX* (**B**) genes at 0, 8, 12, and 48 h after treatment with 200 μM MeJA as determined with qRT-PCR. Expression levels of *MtLOX* and *MsLOX* genes were normalized to levels of *MtActin* and *MsActin*, respectively. Hierarchical gene clustering was performed with the normalization method “standard score” on log_2_ transformed data. Colors indicate changes in expression compared to the 0 h timepoint, with red indicating an increase and blue indicating a decrease in expression

JA is a product of the fatty acid synthesis pathway that is involved in plant growth and development (Albechtová, 1994). To investigate the potential roles of *LOX* genes in response to stress induced by exogenous MeJA treatment (200 µM), the expression levels of 18 *MtLOX* genes and 13 *MsLOX* genes were determined in MeJA-treated plants via qRT-PCR (Fig. 6). Eight *MtLOX* genes (*MtLOX6*, *MtLOX9*, *MtLOX11*, *MtLOX16*, *MtLOX20*, *MtLOX21*, *MtLOX22*, and *MtLOX24*) and seven *MsLOX* genes (*MsLOX18*, *MsLOX23*, *MsLOX30*, *MsLOX84*, *MsLOX85*, *MsLOX90*, and *MsLOX94*) were up-regulated at 8 h of MeJA treatment; *MtLOX16*, *MtLOX20*, *MtLOX21*, *MtLOX22, MsLOX*18, *MsLOX23*, *MsLOX84*, and *MsLOX85* were statistically significantly up-regulated. Three *MtLOX* genes (*MtLOX*2, *MtLOX13*, and *MtLOX26*) and three *MsLOX*s (*MsLOX7, MsLOX14*, and* MsLOX87*) were down-regulated after MeJA exposure.

### Constitutive *MtLOX24* expression in *Arabidopsis* increased tolerance to MeJA stress

**Fig. 7:**
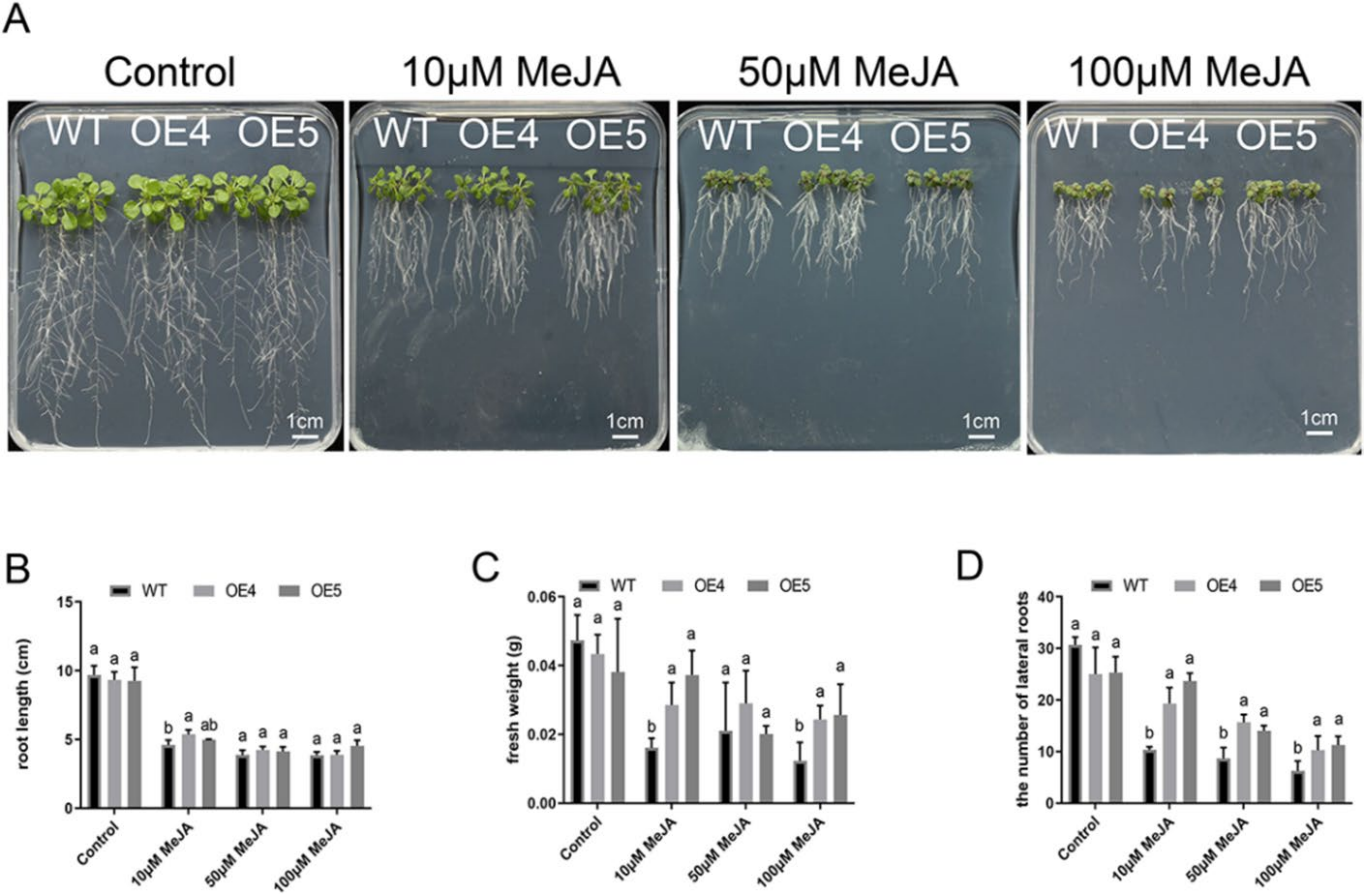
Overexpression of *MtLOX24* resulted in resistance to exogenous methyl jasmonate (MeJA) treatment of *Arabidopsis* seedlings. **A** Root growth among wild-type (WT) *Arabidopsis* seedlings and those overexpressing *MtLOX24* (OE4 and OE5). Seven-d-old seedlings grown on ½ × Murashige and Skoog (MS) medium were transferred to ½ × MS medium supplemented with 0, 50, or 100 μM MeJA. Plants were assessed after two weeks. **B** Quantification of root length. **C** Fresh weight. **D** The number of lateral roots of *Arabidopsis* seedlings. Error bar represents the standard error calculated by three independent experiments

**Fig. 8:**
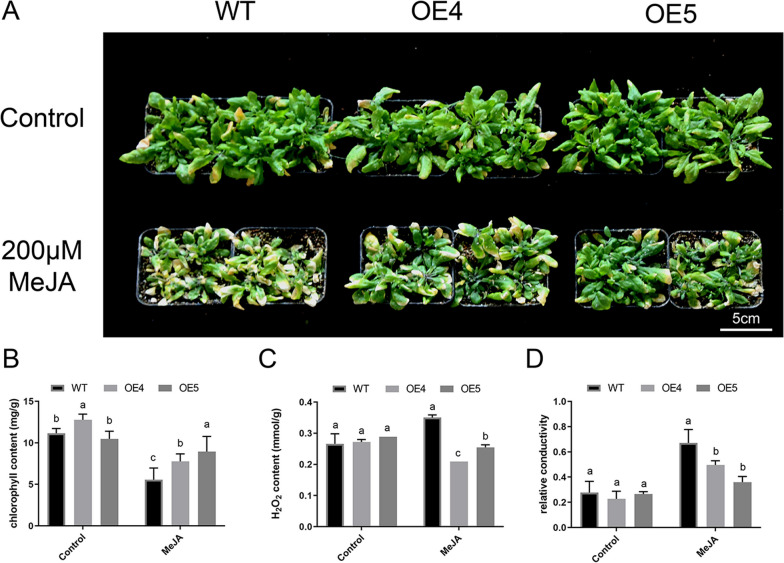
Overexpression of the *Medicago truncatula* gene *LOX24* in *Arabidopsis thaliana* resulted in resistance to exogenous methyl jasmonate (MeJA) treatment among seedlings grown in soil. **A** Phenotypes of wild-type (WT) and *MtLOX24*-overexpression (OE4 and OE5) *Arabidopsis* plants grown in soil with or without MeJA treatment for three weeks. **B** Quantification of chlorophyll content. **C** H_2_O_2_ content. **D** Relative conductivity in *Arabidopsis* seedlings. Error bars represent the standard error calculated from three independent experiments

*MtLOX* gene expression analysis in plants treated with exogenous MeJA (described above) showed that 44.4% of *MtLOX* genes were MeJA-inducible. Because LOXs are involved in JA synthesis, it is important to understand their responses to MeJA treatment. LOXs are also associated with abiotic stress, including insect pest stress (as simulated by MeJA treatment). In this study, we constructed 8 *MtLOX24*-overexpression (OE) lines in *Arabidopsis* and selected two lines (OE4, OE5) for further study. Gene expression level of the overexpression lines was confirmed using qRT-PCR (Additional file 13 Figure S5). In ½ × Murashige and Skoog (MS) medium, the *Arabidopsis* OE lines showed no differences in root length, fresh weight, or lateral root number compared to wild-type (WT) seedlings under normal growth conditions (Fig. 7). However, when 10 μM or 100 μM MeJA was added to the growth medium, the OE lines showed significantly higher fresh weights and lateral root growth than the WT seedlings (Fig. 7A-D). At 50 μM MeJA, the two OE lines had significantly more lateral roots than the WT, but differences in the root lengths and fresh weights were not significant (Fig. 7C-D). The root length-inhibiting effect of MeJA was not stronger in the OE lines than in the WT (Fig. 7B). In a separate experiment, MeJA was applied to three-week-old plants grown in soil; WT plants showed a higher degree of damage than the OE lines, with more yellow leaves (Fig. 8A), decreased chlorophyll content, increased H_2_O_2_ and increased relative conductivity levels (Fig. 8B-D).

## Discussion

The size of the *LOX* gene family varies greatly between species; there are 95 family members in *M. sativa*, 40 in *G. max*, 30 in *M. truncatula*, and only six in *A. thaliana*. These variations are largely consistent with differences in plant genome size: 2.74 Gb for alfalfa cv. ‘ZM-4’, 994 Mb for *G. max*, 450–500 Mb for *M. truncatula*, and -135 Mb for *Arabidopsis* [39–41]. Phylogenetic analysis has previously shown that the *LOX* family can be divided into two subfamilies, 9-LOX and 13-LOX. In *M. truncatula* and *M. sativa*, the 13-LOX subfamily can be further divided into two subfamilies: type I and type II 13-LOX. Evolutionary analysis here showed that *GmLOX* and *MtLOX* genes clustered together within each subfamily. This may be because *GmLOX* and *MtLOX* have a common ancestor [42]. Most *MtLOX* genes clustered together with three to five *MsLOX* genes each, indicating that numerous *MsLOX* genes may have arisen from gene duplication events after speciation. The type I 13-LOX cluster included the largest number of *MsLOX* genes. In this subfamily, only *Glyma.15G026300* clustered together with the *MtLOX*s and *MsLOX*s, and the other seven *GmLOX* genes clustered together into a separate clade. This suggested that type I 13-LOX genes were significantly different in *Medicago* species compared to those in soybean. However, there were more 9-LOX and type II 13-LOX genes in *G. max* than in *M. truncatula*. This may be because the *GmLOX* family expanded as a result of ancient polyploidization events prior to speciation, leading to the emergence of soybean-specific gene duplicates [42].

As part of *MtLOX* and *MsLOX* characterization, we predicted subcellular localization. The 9-LOX and type I 13-LOX proteins in *M. truncatula* and *M. sativa* had no transport peptides, and most were therefore predicted to be localized to the cytoplasm. The exceptions were MtLOX21, MsLOX52, MsLOX53, MsLOX65, and MsLOX85, which were predicted to be localized only to the chloroplasts. These results were consistent with previous findings in rice [43] and banana [20]. Other proteins were predicted to be localized to both the cytoplasm and the chloroplasts; these included four MtLOXs (MtLOX2, MtLOX15, MtLOX17, and MtLOX18) and 13 MsLOXs (MsLOX5, MsLOX8, MsLOX12, MsLOX14, MsLOX51, MsLOX54, MsLOX56, MsLOX70, MsLOX73, MsLOX77, MsLOX82, MsLOX86, and MsLOX92). In addition to MsLOX4, MsLOX27, MsLOX31, and MsLOX40, all type II 13-LOX proteins in *M. truncatula* and *M. sativa* contained chloroplast transport peptides, and MtLOX6 and MsLOX32 were predicted to be localized to both the cytoplasm and the chloroplasts. This may have been due to sequences in these genes encoding chloroplast transport peptides, and the sequences have originated from different exons [44]. Type II 13-LOX genes localized to the chloroplasts may be involved in JA biosynthesis and resistance to insect herbivory, as previously reported for the tomato (*Solanum lycopersicum*) protein TomLoxD [45]. MsLOX27 and MsLOX31 were predicted to be localized to the mitochondria. LOXs in plant mitochondria may mediate the degradation of key membrane phospholipids and fatty acids [46].

*Cis*-acting regulatory elements are common among the promoters of stress-responsive genes. Identification and functional verification of such elements can aid in understanding transcriptional regulation of genes throughout the genome. Several phytohormone-responsive elements have been well characterized in plants. For example, the *cis*-element ABRE (PyACGTGGC) [47] was found in the promoter of the *Arabidopsis* dehydration-responsive gene *RD29B*, which is involved in transcriptional activation in response to ABA [48]. CGTCA-motifs are associated with MeJA responses, and TCA-elements are found in genes that participate in SA-related responses. Other *cis*-elements are associated with abiotic and biotic stress signaling. For example, the ARE element is found in antioxidant genes that can enhance environmental carcinogen detoxification [49]. In *Neurospora crassa*, the STRE motif is involved in regulation of a variety of cellular processes, including stress responses, carbohydrate metabolism restriction, and ethanol tolerance (through binding to Msn2p and Msn4p) [50, 51]. We here found numerous instances of the motifs mentioned above in *MtLOX* and *MsLOX* genes. Some genes, such as *MtLOX5*, *MtLOX6*, *MtLOX8*, *MtLOX20*, *MtLOX23*, *MsLOX4*, *MsLOX15*, *MsLOX17*, *MsLOX43*, and *MsLOX94*, contained multiple ABRE and CGTCA-motif elements. This suggested that these genes may play crucial roles in plant hormone regulation. The GT1-motif has often been studied as a light-responsive element; under light conditions, this motif is bound by the activation domain factor P, activating transcription [52]. The GT1-motif and the WUN-motif, the latter of which is associated with circadian rhythm, were found in the promoters of *MtLOX1*, *MtLOX18*, *MtLOX24*, *MtLOX26*, *MsLOX55*, *MsLOX82*, and *MsLOX94*, indicating that these genes may be light responsive. Few MBS and LTR motifs were found in *MtLOX*s and *MsLOX*s, likely because these elements are most often found in animal cell viruses, such as avian myeloblastosis virus [53] and avian leukosis virus [54].

*LOX* family genes show extensive spatiotemporal expression variation; these genes are involved in seed germination [55], seedling growth, and plant development and senescence [56]. Here, qRT-PCR showed that some *MtLOX* genes were highly expressed in the flowers and stems, suggesting that several *MtLOX* family genes played common roles in reproductive growth in *M. truncatula*. Six of the genes with high expression in the flowers, excluding *MtLOX7* and *MtLOX11*, belonged to the type I 13-LOX subfamily, suggesting that type I 13-LOX genes play important roles in *M. truncatula* flower development. Other *MtLOX* genes, such as *MtLOX11*, also likely participate in flower and organ development. In contrast, in *M. sativa*, *MsLOX* genes from different subfamilies (e.g., *MsLOX23, MsLOX87, MsLOX90,* and *MsLOX94*) were highly expressed in the flowers. In *Arabidopsis*, the 13-LOX genes *AtLOX3* and *AtLOX4* have roles in plant vegetative growth and flower development [57]. In tea (*Camellia sinensis*), *LOX1* is primarily expressed in the flowers, and is up-regulated during petal senescence and down-regulated during the bud opening period [56]. Analysis of previously published *MtLOX* expression data showed that they were highly expressed in the leaves, petioles, stems, VegBuds, flowers, and pods, but that some 9-LOXs and type I 13-LOXs (such as *MtLOX12*, *MtLOX15*, *MtLOX19*, *MtLOX20*, *MtLOX21*, *MtLOX22*, and *MtLOX28*) participated in root nodule synthesis and were induced by arbuscular mycorrhizal fungi. Similar results have also been reported in tomatoes. LOX activity is induced by inoculation with pathogens such as *Funneliformis mosseae*, *Alternaria solani*, and *Cladosporium fulvum* in tomato [58, 59], and *C. fulvum* tolerance is increased by *TomloxD* overexpression [24]. In *Vicia faba* [60], *Phaseolus vulgaris* [61], and *G. max* [62], the *LOX* genes *VfLOX1*, *PvLOX5*, and *GmLOX1*, respectively, show consistent expression levels during nodule development, suggesting that *LOX* genes are involved in the symbiotic nitrogen fixation process between rhizobia and legumes.

Lipoxygenase functions in both herbivore feeding and JA treatment via the octadecanoic acid pathway [45]. In *Arabidopsis*, potato, and tobacco (*Nicotiana attenuata*), silencing the 13-LOX gene *LOX2* [63], a 13-LOX isoform (LOX-H3) [64], or *LOX3* [65], respectively, decreases the tolerance of the plant to insect-induced stress. Analysis of *LOX* expression in response to MeJA exposure in both *M. truncatula* and *M. sativa* showed differences between *LOX* subfamilies in this study. Specifically, 9-LOX subfamily genes showed little change in expression levels after MeJA treatment. However, type I and II 13-LOX genes were strongly induced by MeJA in both species, suggesting that they may promote insect resistance by participating in the JA signaling pathway. Specifically, *MtLOX5*, *MtLOX16*, *MtLOX20*, *MtLOX21*, and *MtLOX22* were up-regulated by 6–53 times at 8 h of MeJA treatment; *MtLOX6*, *MtLOX9*, *MtLOX10*, and *MtLOX24* were induced by two to four times at the same timepoint. Similarly, *MsLOX18*, *MsLOX23*, *MsLOX84*, and *MsLOX85* were induced by 4–47 times, whereas *MsLOX30*, *MsLOX57*, *MsLOX90*, and *MsLOX94* were up-regulated by 1.5–3 times at 8 h after MeJA treatment. In our study, it was found that overexpression of the *MtLOX24* in *Arabidopsis* could alleviate the damage caused by MeJA treatment, which may be due to the fact that *MtLOX24*, as a member of the 13-LOX gene subfamily, can catalyze α-LeA to produce 13-HPOT and increase the content of endogenous JA, which needs to be verified in subsequent studies.

## Conclusion

In this study, a total of 30 *MtLOX* and 95 *MsLOX* genes were identified. *LOX* genes can be further divided into three subfamilies, 9-LOX, type I 13-LOX, and type II 13-LOX. Promoter element analysis predicted that LOXs were involved in abiotic stress response, hormone response and plant development. The expression profiles of *MtLOX* and *MsLOX* showed that the expression of 13-LOXs was different in different organs, and the expression of 13-LOXs was induced by MeJA. In addition, *Arabidopsis* overexpressing *MtLOX24* showed resistance to exogenous MeJA and alleviated the oxidative damage of *Arabidopsis* leaves induced by MeJA, which may be involved in JA signaling pathways. In conclusion, this study provides important information on the potential function of alfalfa LOXs.

## Methods

### Plant materials, growth conditions, and stress treatments

*M. truncatula* R108 and *M. sativa* Zhongmu No. 4 were used in this study. Tetraploid alfalfa cultivar Zhongmu No. 4 was bred by the author Qingchuan Yang. Diploid *M. truncatula* ecotype R108 was provided by the Samuel Roberts Noble Foundation (U.S.A.) and preserved by our laboratory. The procedures of plant material collection complied with relevant institutional, national, and international guidelines and legislation. Sterilized seeds were germinated in petri dishes covered with double layer of filter paper. Five days after germination, seedlings were transferred to ½ × Hoagland solution for subsequent growth. For stress treatments, after growth for 3 weeks, MeJA was added to a final concentration of 200 µM. Samples were taken at 0, 8, 12, and 48 h after treatment. The stems, leaves, and flowers were collected for differential gene expression analysis. Plants were grown at 25/22 ℃ under a 16/8 h light/dark cycle with 60% relative humidity.

### Genome-wide identification of *LOX* genes

LOX protein sequences for *Arabidopsis*,* M. truncatula*, and* G. max* were obtained from Phytozome (https://phytozome.jgi.doe.gov/pz/). The gene files for the ZM-4 alfalfa genome were downloaded from the following site: https://figshare.com/s/fb4ba8e0b871007a9e6c. The LOX domain (PF00305) was downloaded from Pfam (http://pfam.xfam.org/), and the Hidden Markov Model (HMM) Profile model was used to identify potential *LOX* family genes. A total of 95 putative LOX proteins were identified in the alfalfa ZM-4 genome that contained both PLAT (PF01477) and LOX (PF00305) domains, and these were selected as predicted *MsLOX* genes. ProtParam (https://web.expasy.org/protparam/) was used to analyze predicted MtLOX and MsLOX physical and chemical properties, including molecular weight, theoretical isoelectric point, and GRAVY values. Subcellular localization of each protein was predicted with Cell-PLoc 2.0 (http://www.csbio.sjtu.edu.cn/bioinf/Cell-PLoc-2/, accessed on 25 February 2021).

### Analyses of phylogenetic relationships, gene structures, and conserved sequences

We used 171 full-length *LOX* gene sequences in the phylogenetic analysis, comprising six *AtLOX*s, 40 *GmLOX*s, 30 *MtLOX*s, and 95 *MsLOX*s. All sequences were aligned using the EMBL-EBI multiple sequence alignment tool (https://www.ebi.ac.uk/Tools/msa/). A phylogenetic tree was then constructed and visualized with the Interactive Tree Of Life (http://itol.embl.de/) using the default settings.

The *LOX* sequences from *M. truncatula* and *M. sativa* were aligned in MEGA5 [66] and a phylogenetic tree was constructed using the neighbor-joining (NJ) method with default parameters and 1000 bootstrap replicates. The conserved motifs of MtLOX and MsLOX proteins were identified with the MEME suite (http://meme-suite.org/tools/meme). Protein domain content was predicted with NCBI CDD (https://www.ncbi.nlm.nih.gov/Structure/bwrpsb/bwrpsb.cgi). The intron–exon structures of *MtLOX*s and *MsLOX*s were determined using the Gene Structure Display Server (http://gsds.cbi.pku.edu.cn). The final gene structures and conserved sequences were visualized with TBtools software [67].

### Chromosome location analysis and protein–protein interaction network prediction

The general feature format (gff) files for *MtLOX* genes were downloaded from Ensembl plants (http://plants.ensembl.org/index.html) and the gff files for the alfalfa genome were downloaded from the following site: https://figshare.com/s/fb4ba8e0b871007a9e6c. *M. truncatula* and *M. sativa* genome length and gene mapping information were extracted with Tbtools [67]. A physical chromosomal location map was drawn with MapGene2Chrom web v2 (http://mg2c.iask.in/mg2c_v2.0/). The transcript identification numbers of *MtLOX* genes (Additional file 1: Table S1). The STRING database (https://cn.string-db.org/) was used to predict interactions among 14 MtLOXs using a protein–protein interaction (PPI) enrichment threshold of *p* < 1.0e-16.

### *Cis*-acting regulatory element analysis

The PlantCARE database (http://bioinformatics.psb.ugent.be/webtools/plantcare/html/) was used to predict *cis*-elements in the 2-kb promoter region upstream of the transcription start site of each *MtLOX* and *MsLOX* gene. The results were visualized with TBtools software [67].

### Expression patterns of *LOX *genes in *M. truncatula* based on transcriptomic data

Expression data were obtained from the *M. truncatula* Gene Expression Atlas (https://medicago.toulouse.inrae.fr/MtExpress) for leaves, petioles, stems, VegBuds, flowers, pods, roots, seeds, nodules at multiple stages (Nod_4dpi and Nod_14dpi), RT_LCM_adjacent, RT_LCM_arbuscular, and RT_LCM_cortical. The sampling methods are described in detail by Benedito [68]. Dry mature seeds (DS) were collected from developing *M. truncatula*; the seeds were removed from the pods at 48-d intervals [69]. Transcripts of LCM-derived arbuscule-containing cells from mycorrhizal roots (RT_LCM_arbuscular), cortical cells of non-mycorrhizal roots (RT_LCM_cortical), and cortex cells adjacent to extracellular fungal hyphae (RT_LCM_adjacent) were obtained from *Medicago* (A17) genome arrays [70]. The expression data were log_2_ transformed [71] and visualized as heatmaps with TBtools.

### Expression patterns of *MtLOX* and *MsLOX* genes based on qRT‑PCR

*M. truncatula* R108 and alfalfa ‘Zhongmu No. 4’ were treated with MeJA as described above. To analyze *LOX* expression patterns in response to this stress treatment, RNA was extracted from the stems, leaves, and flowers using the Eastep Total RNA Extraction Kit (Promega, Beijing, China). cDNA was generated using the PrimeScript™ RT Reagent Kit with gDNA Eraser (Takara, Shiga, Japan). qRT-PCR was conducted on the CFX384 Touch Real-time PCR Detection System (Bio-Rad, Hercules, CA, USA). The *MtActin* and *MsActin* genes were used as the internal references for *MtLOX*s and *MsLOX*s, respectively, and expression levels were normalized using the 2^−ΔΔCt^ method (Livak and Schmittgen, 2001). The expression data were then log_2_ transformed [71] and visualized as heatmaps with TBtools. All primers used in this study were designed using NCBI Primer-Blast (nih.gov) and are shown in Additional file 12: Table S8.

### Exogenous *MtLOX24* expression in *Arabidopsis*

*MtLOX24* (Medtr8g018690) was amplified from *M. truncatula* cDNA with the following oligonucleotide primers: 5′-GAAGTGAGCAAGCCACAAGC-3′ and 5′-ACATAGGAGGATGAGGGAAT-3′. The *MtLOX24* coding sequence was inserted into the pCAMBIA3301 vector under the control of the CaMV 35S promoter. The resulting 35S::*MtLOX24*-GUS expression plasmid was transformed into *Arabidopsis* ecotype Columbia 0 using the floral dip method [72]. T_3_ homozygous plants were used in further experiments. Seeds were held at 4 ℃ in the dark for 2 d, then cultured on ½ × Murashige and Skoog (MS) medium in an incubator under a 16/8 h light/dark photoperiod at 22/18 ℃. After 7 d, seedlings were transferred to ½ × MS medium supplemented with MeJA (0, 50, or 100 µM) for 15 d. The fresh weight, root length, and lateral root number were then measured. Three-week-old transgenic *Arabidopsis* plants were also grown in soil treated with 0 or 200 µM MeJA solution every 3 d for a period of three weeks. For physiological index measurements, the chlorophyll content was measured according to Ergun [73], relative conductivity was mainly used to study the change trend of membrane permeability and were detected by method as described by Zhou B [74]. The concentration of H_2_O_2_ was determined according to the protocol of the H_2_O_2_ detection kit provided by the manufacturer (Nanjing JianchengInstitute of Bioengineering).

### Statistical analysis

SPSS software was used for statistical analysis. The mean and standard deviation were calculated from three independent experiments with comparable results. All data collected were analysed using one-way ANOVA and Duncan's multiple mean comparison test was used to compare the control and treatment groups.

### Supplementary Information

**Additional file 1. Table S1.** Properties of *LOX* gene family in *Medicago truncatula*.

**Additional file 2. Table S2.** Properties of *LOX* gene family in *Medicago sativa*.

**Additional file 3. Figure S1.** Chromosomal locations of *LOX* genes in *Medicago truncatula* and *Medicago sativa*.

**Additional file 4. Table S3.** Members of the *LOX* gene family in *Medicago truncatula.*

**Additional file 5. Table S4.** Members of the *LOX* gene family in *Medicago sativa.*

**Additional file 6. Figure S2.** Conserved residues in *LOX* family proteins.

**Additional file 7. Table S5.** Conserved structural domains in *Medicago truncatula* LOXs.

**Additional file 8. Table S6.** Conserved structural domains in *Medicago sativa* LOXs.

**Additional file 9. Figure S3.** Protein-protein interaction network analysis.

**Additional file 10. Figure S4.** Expression profiles of *Medicago truncatula* LOX genes in multiple plant organs and under several treatment conditions.

**Additional file 11. Table S7.** *Medicago truncatula* *LOX* gene expression profiles. Data were downloaded from the *M. truncatula* Gene Expression Atlas.

**Additional file 12. Table S8.** Primers used in this study.

**Additional file 13. Figure S5.** The relative expression level of *MtLOX* from overexpressed *Arabidopsis* lines using qRT-PCR.

## Data Availability

The transcriptomic data were obtained from the *M. truncatula* Gene Expression Atlas (https://medicago.toulouse.inrae.fr/MtExpress).
