# Supplementary material for: A genome-wide study of the lipoxygenase gene families in Medicago truncatula and Medicago sativa reveals that MtLOX24 participates in the methyl jasmonate response
Source: BMC Genomics. 2024 Feb 19;25:195. doi: 10.1186/s12864-024-10071-1 (PMC10875803; doi:10.1186/s12864-024-10071-1)
Supplement: Supplementary file 3 — Additional file 3. Figure S1. Chromosomal locations of LOX genes in Medicago truncatula and Medicago sativa. [file 12864_2024_10071_MOESM3_ESM.docx]

*
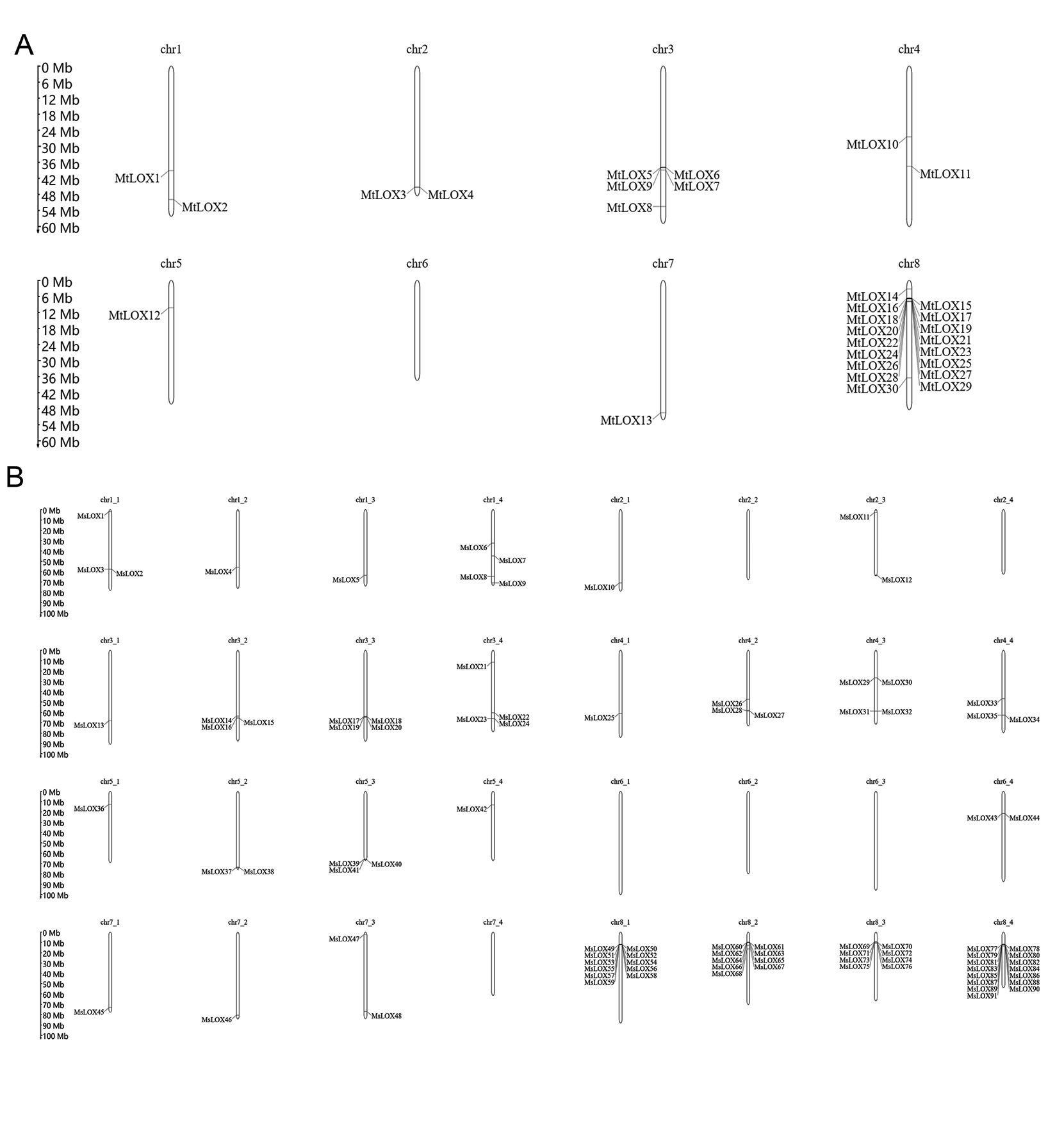
*

**Figure S1.** Chromosomal locations of *LOX* genes in *Medicago truncatula* and *Medicago sativa*. Chromosomal locations of 30 *MtLOX* genes **(A)** and 95 *MsLOX* genes **(B)**.
