# Supplementary material for: A genome-wide study of the lipoxygenase gene families in Medicago truncatula and Medicago sativa reveals that MtLOX24 participates in the methyl jasmonate response
Source: BMC Genomics. 2024 Feb 19;25:195. doi: 10.1186/s12864-024-10071-1 (PMC10875803; doi:10.1186/s12864-024-10071-1)
Supplement: Supplementary file 6 — Additional file 6. Figure S2. Conserved residues in LOX family proteins. [file 12864_2024_10071_MOESM6_ESM.docx]

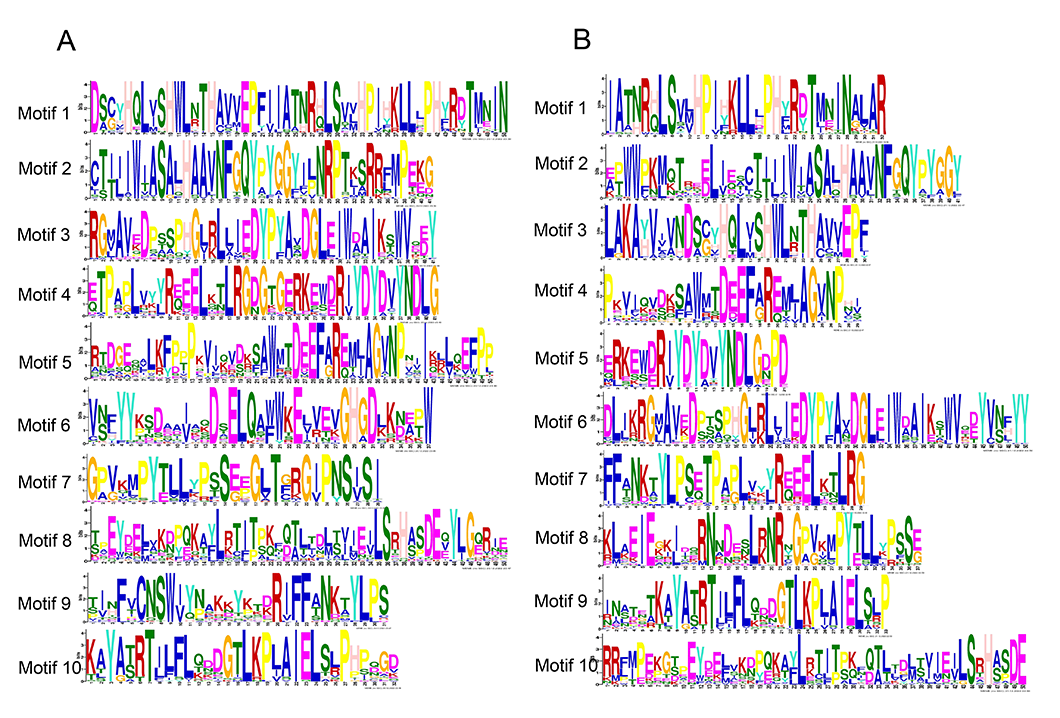


**Figure S2.** Conserved residues in *LOX* family proteins. Conserved residues were identified in LOX proteins in *Medicago truncatula* **(A)** and *Medicago sativa* **(B)** with the MEME suite (http://meme-suite.org/tools/meme).
