# Supplementary material for: A genome-wide study of the lipoxygenase gene families in Medicago truncatula and Medicago sativa reveals that MtLOX24 participates in the methyl jasmonate response
Source: BMC Genomics. 2024 Feb 19;25:195. doi: 10.1186/s12864-024-10071-1 (PMC10875803; doi:10.1186/s12864-024-10071-1)
Supplement: Supplementary file 9 — Additional file 9. Figure S3. Protein-protein interaction network analysis. [file 12864_2024_10071_MOESM9_ESM.docx]

**
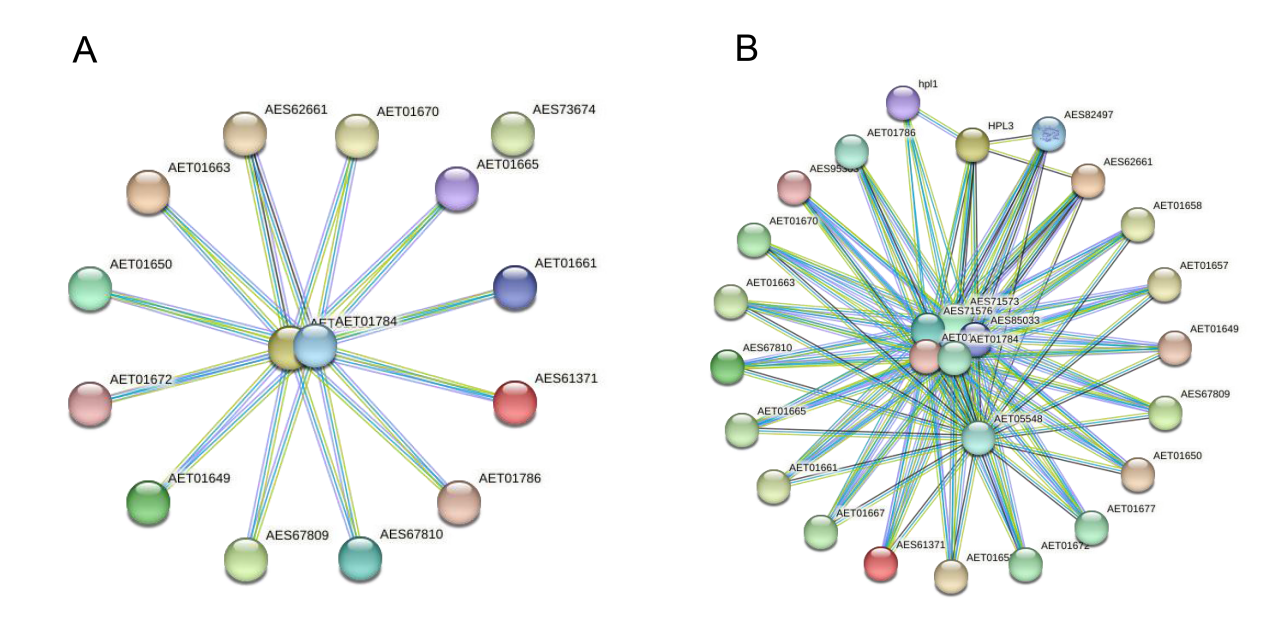
**

**Figure S3.** Protein-protein interaction network analysis. Interactions among 14 Medicago truncatula LOXs **(A)** and 21 Medicago sativa LOXs **(B)** . Protein interactions were predicted with the STRING database.
