# Supplementary material for: A genome-wide study of the lipoxygenase gene families in Medicago truncatula and Medicago sativa reveals that MtLOX24 participates in the methyl jasmonate response
Source: BMC Genomics. 2024 Feb 19;25:195. doi: 10.1186/s12864-024-10071-1 (PMC10875803; doi:10.1186/s12864-024-10071-1)
Supplement: Supplementary file 10 — Additional file 10. Figure S4. Expression profiles of Medicago truncatula LOX genes in multiple plant organs and under several treatment conditions. [file 12864_2024_10071_MOESM10_ESM.docx]

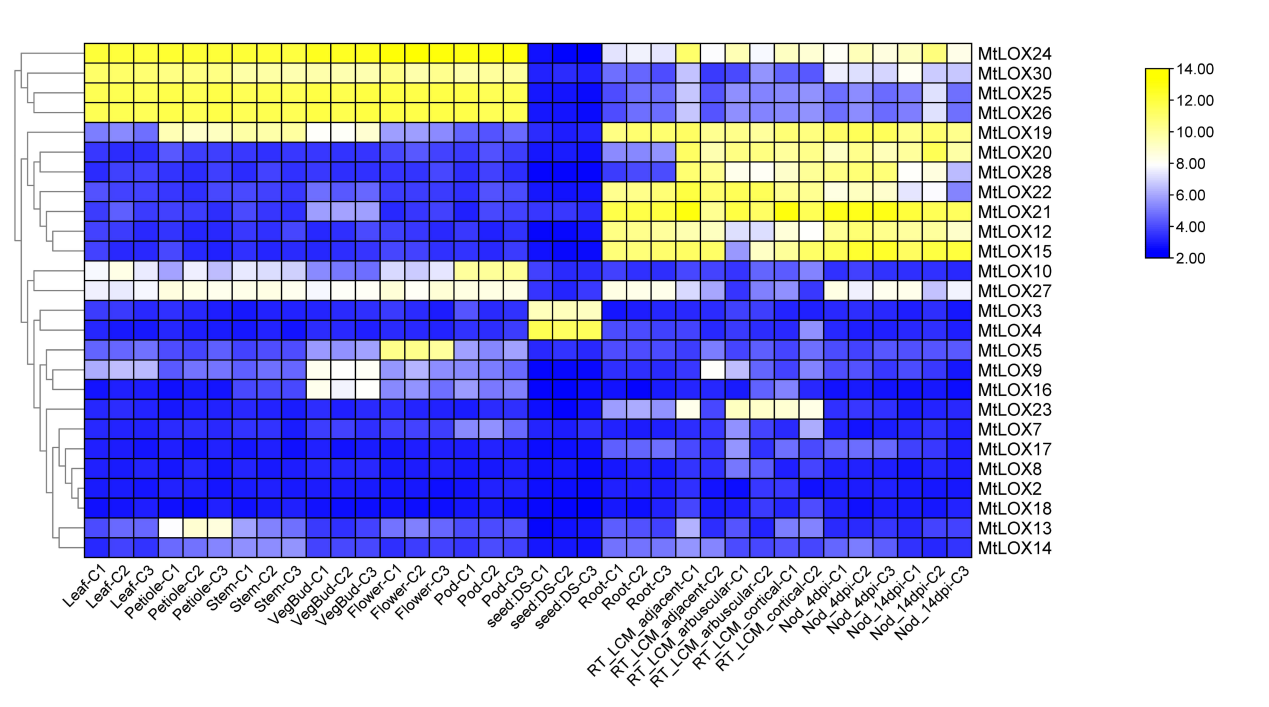


**Figure S4.** Expression profiles of *Medicago truncatula LOX* genes in multiple plant organs and under several treatment conditions. The transcriptomic data were obtained from the *M. truncatula* Gene Expression Atlas (https://medicago.toulouse.inrae.fr/MtExpress). The heatmap showing gene expression data was generated with TBTools.
