# Supplementary material for: A genome-wide study of the lipoxygenase gene families in Medicago truncatula and Medicago sativa reveals that MtLOX24 participates in the methyl jasmonate response
Source: BMC Genomics. 2024 Feb 19;25:195. doi: 10.1186/s12864-024-10071-1 (PMC10875803; doi:10.1186/s12864-024-10071-1)
Supplement: Supplementary file 13 — Additional file 13. Figure S5. The relative expression level of MtLOX from overexpressed Arabidopsis lines using qRT-PCR. [file 12864_2024_10071_MOESM13_ESM.docx]

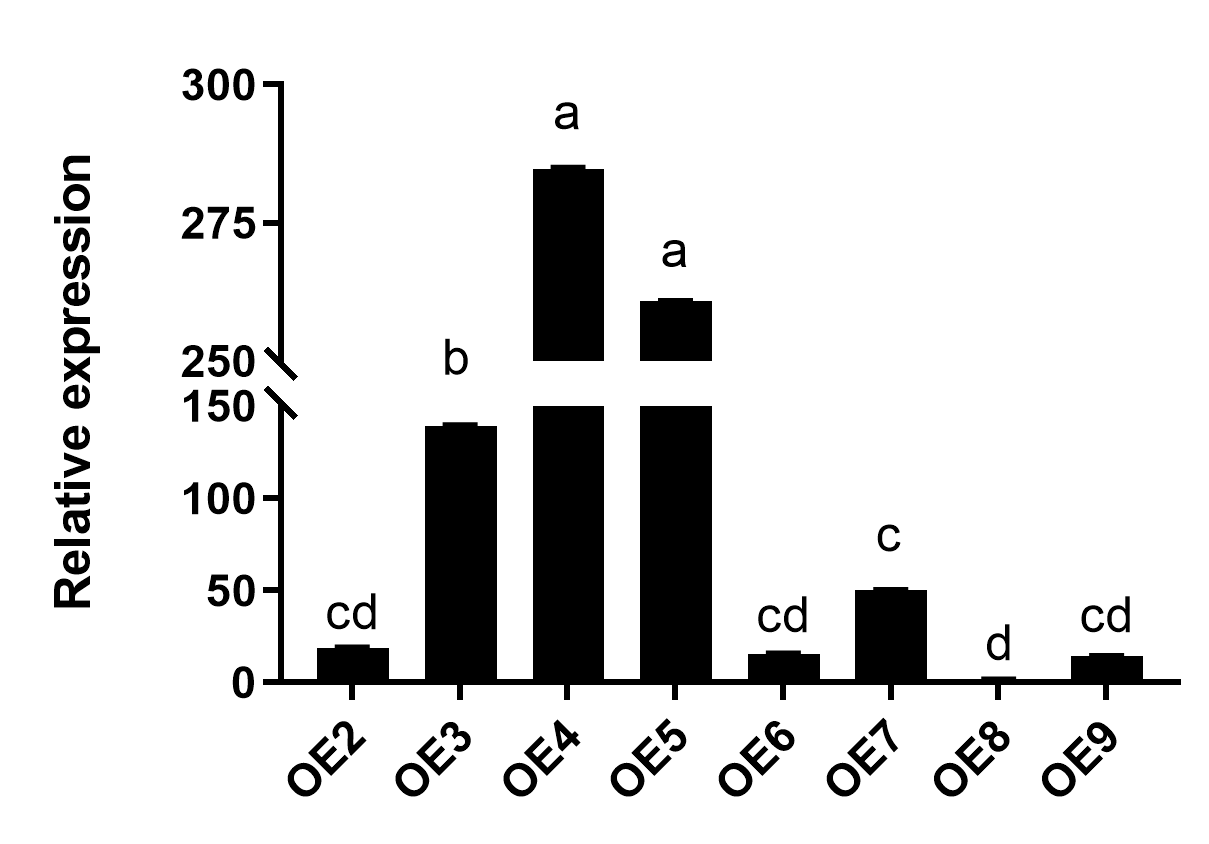


**Figure S5.** The relative expression level of *MtLOX* from overexpressed *Arabidopsis* lines using qRT-PCR.
